# Cutaneous Tattoo Ink as a Mimicker of Endometriosis-Like Lesions on Diagnostic Laparoscopy

**DOI:** 10.7759/cureus.59212

**Published:** 2024-04-28

**Authors:** Andrew M Tannous, Brittney D Bastow

**Affiliations:** 1 Obstetrics and Gynecology, Saint Joseph Hospital, Denver, USA; 2 Obstetrics and Gynecology, Kaiser Permanente, Denver, USA

**Keywords:** women’s health, chronic pelvic pain, diagnostic laparoscopy, tattoo ink, endometriosis

## Abstract

This paper presents the case of a 28-year-old with a history of chronic pelvic pain suspicious of endometriosis. She underwent a diagnostic laparoscopy and biopsy of lesions along the posterior cul-de-sac and right sidewall near the external iliac artery. Histopathological examination revealed no evidence of endometriosis but did comment on benign lymph node tissue with tattoo-like pigment. These findings correspond to the patient’s tattoos located along the lower extremities. It is suspected there was cutaneous tattoo ink migration causing intra-abdominal lymphadenopathy, which visually mimicked endometriosis on diagnostic laparoscopy. Surgeons should become familiar with and recognize this phenomenon, as it can be misleading in the setting of endometriosis and diagnostic laparoscopy. Surgeons undertaking these cases must be able to identify and safely excise abnormal-appearing lesions in many different locations to prevent a missed or delayed diagnosis of endometriosis.

## Introduction

Endometriosis affects roughly 10% of reproductive-age women globally, or upwards of 190 million women worldwide [1]. Given that the diagnosis of endometriosis requires histopathological analysis, the gynecological surgeon must use their best clinical judgment for safe intraoperative excision. This can be challenging to biopsy or remove lesions on areas such as the ovary, fallopian tubes, bladder, intestines, and areas adjacent to major blood vessels and nerves within the pelvis.

Tattooing among women has gained increasing visibility and popularity in Western countries. It is currently estimated that 10-20% of US women have a tattoo [2]. Histologically, tattooing creates an acute inflammatory reaction and gradual assimilation of ink into macrophages [3]. Eventually, much of the pigment is carried to the regional draining lymph nodes and causes reactive lymphadenopathy, which can lead to difficulty in identifying and diagnosing medical issues [4]. This phenomenon has been documented in the setting of sentinel lymph node dissection for detecting breast malignancy, melanoma, and vulvar and cervical cancers [4-11]. There is a known case of colonoscopic tattoo dye spillage that was found to mimic intra-peritoneal endometriosis on laparoscopy [12]. However, to our knowledge, there is no known literature documenting reactive lymphadenopathy to cutaneous tattoo pigment in the setting of suspected endometriosis and tissue biopsy during diagnostic laparoscopy. 

Here, we present a case of chronic pelvic pain in which a diagnostic laparoscopy was performed for clinically presumed endometriosis. However, on histopathologic assessment of intra-abdominally excised tissue, a particular lesion that appeared to be suspicious for endometriosis on laparoscopy was the result of reactive changes to tattoo pigment migration.

## Case presentation

A 28-year-old woman, Gravida 0, presented with a six-month history of worsening dysmenorrhea. Her medical history was significant for constipation-predominant irritable bowel syndrome (IBS). She reported this pain was distinct from the pain associated with her IBS, which has been co-managed by her gastroenterologist and primary care provider since she was diagnosed in 2014. She described very painful but light monthly menses. Over months, her pain became constant, and she could no longer discern any temporal association with her menses. She frequently missed work due to the pain. Her pain was exacerbated by bowel movements. Her symptoms were slightly relieved with ibuprofen, stool softeners, and the near-constant application of heating pads. She also received injections of toradol once per week for IBS, which provided minimal relief. 

She had a Kyleena intrauterine device placed four years before her evaluation in a chronic pelvic pain clinic. She had previously used oral contraceptive pills (OCP) but was unsure if it helped her pain. She was sexually active with one male partner. Her most recent cervical cancer screening cytology in August 2021 was negative for intraepithelial lesions or malignancies, with negative co-testing for human papillomavirus (HPV). She had a history of emotional abuse from a prior partner but denied any physical or sexual abuse. She had no history of fibromyalgia. She denied other genitourinary or gastrointestinal symptoms such as dyspareunia, dysuria, frequency, or diarrhea. 

Her abdominal exam was remarkable for tenderness to light and deep palpation in her lower abdomen bilaterally without any musculoskeletal-specific pain. Her pelvic exam showed normal external female genitalia with intact vulvar sensation and reflexes and no signs of vulvodynia. The digital exam was remarkable for severe tenderness in her pelvic floor muscles, including her transverse perineal muscle, bilateral levator ani, and bilateral obturators. No palpable nodularity was appreciated on examination of her bilateral uterosacral ligaments. A bimanual exam demonstrated a small, anteverted, mobile uterus without cervical motion tenderness. Her prior workup included an ultrasound study, which was unremarkable.

Following the patient’s initial assessment, her working diagnosis was chronic pelvic pain, suspected endometriosis, and pelvic floor muscle dysfunction. The patient was conservatively managed with Norethindrone Acetate 2.5mg daily for ovulatory and menstrual suppression, scheduled ibuprofen, multiple neuropathic pain modulators, and vaginal valium. Pelvic floor physical therapy and education on coping with chronic pain were also provided. Despite compliance with conservative management (per review of the chart), the patient reported minimal improvement in her symptoms. During her pelvic pain-focused care, she continued to see her gastroenterologist and primary care physician for IBS.

**Figure 1:**
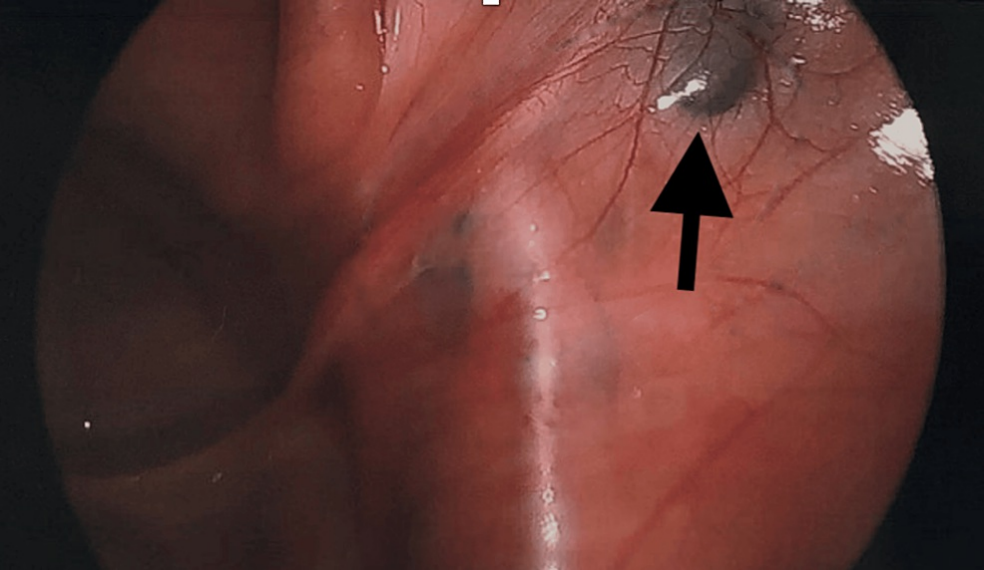
A dark lesion (arrow) resembling gunpowder along the right sidewall

**Figure 2:**
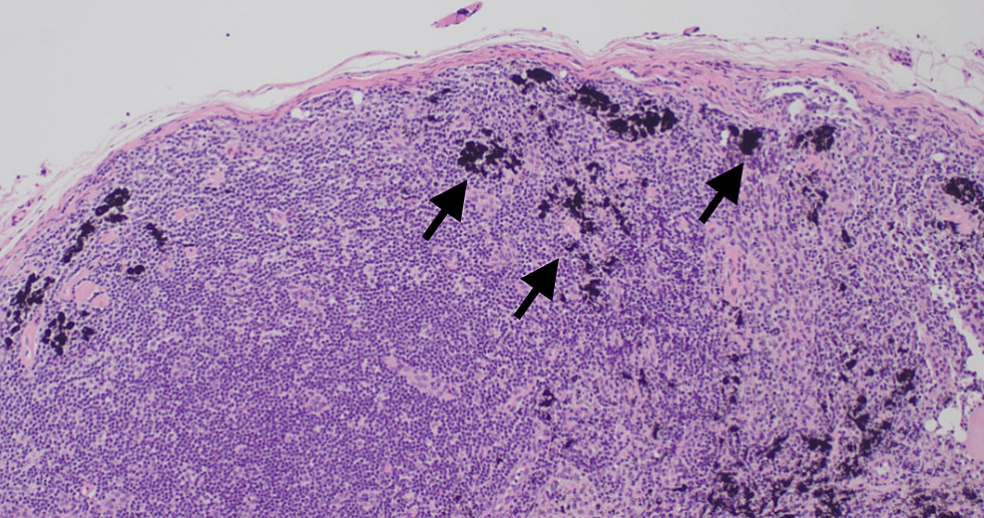
Pigment (arrow) is predominantly intracellular and present in the cytoplasm of histiocytes (20X)

**Figure 3:**
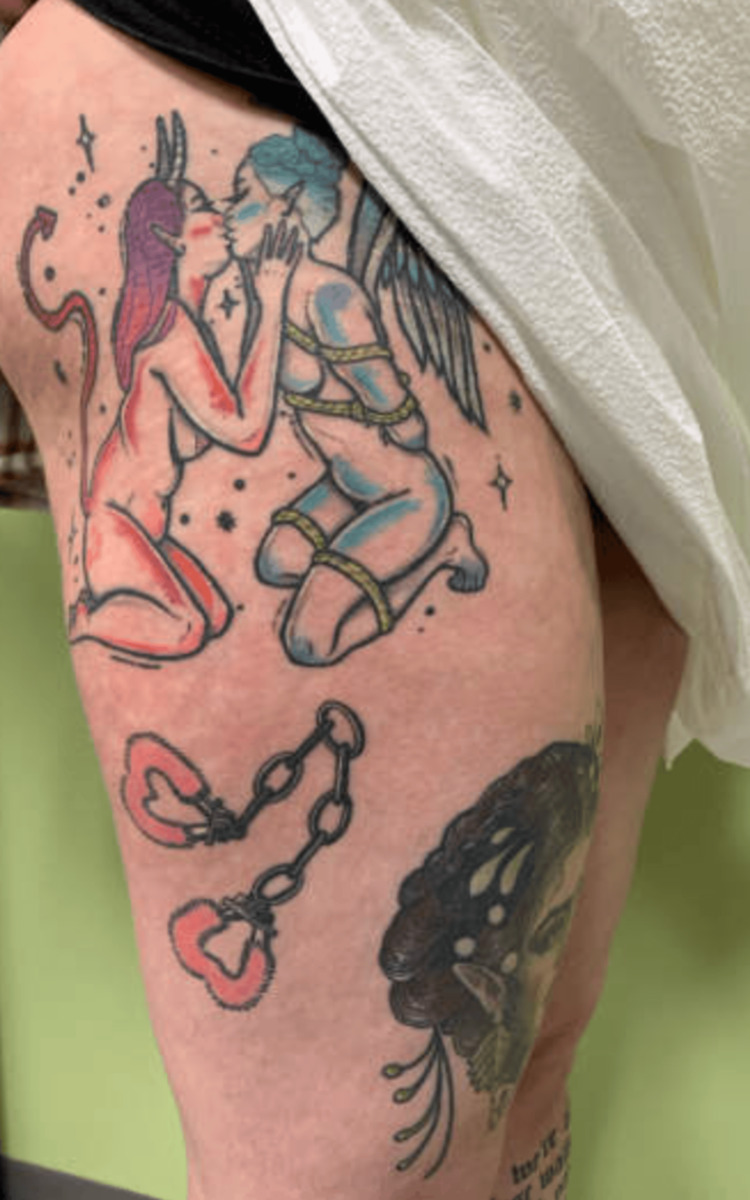
Anterolateral view of the right lower extremity displaying multiple patient tattoos

Given a presumed diagnosis of endometriosis, the decision was made to proceed with an exam under anesthesia, diagnostic laparoscopy, possible excision of lesions, and botox trigger point injections of her pelvic floor muscles. Her exam under anesthesia demonstrated a small, anteverted, mobile uterus. Operative findings were remarkable for two areas of nodularity on the right pelvic sidewall (Figure 1). The nodules were not attached to the overlying peritoneum and were near her iliac vessels. One was able to be excised, but the other lesion was not biopsied due to its proximity to the vasculature. Final pathologic analysis revealed no evidence of endometriosis but did comment on a portion of benign lymph node with tattoo-like pigment belonging to the right sidewall nodule biopsy (Figure 2). The pigment is thought to be attributable to drainage from the regional tattoos, located on the patient’s lower extremities (Figure 3). 

The patient's postoperative course was uncomplicated. She was discharged home in stable condition with close follow-up. She was counseled about her lack of endometriosis on biopsy specimens and was understandably frustrated to not have a formal diagnosis. She continued menstrual suppression, but her chronic pain treatment focused on treating her pelvic floor muscle dysfunction with physical therapy and managing her IBS with a combined approach from primary care, gynecology, and gastroenterology. Further consultation with rheumatology, hematology, and psychiatry was discussed as adjuncts to uncovering the etiology of an additional disease process as well.

## Discussion

Lymphadenopathy is a known complication of tattoo ink migration. Tattooing involves the repeated act of using an ink-filled needle to pierce the epidermis and superficial layer of the dermis. Ink is composed of pigment and carrier solvent, which can be any combination of water, ethanol, formaldehyde, methanol, or antifreeze [13]. Any of these substances can illicit an acute phase reaction in the surrounding phagocytes, which actively migrate the pigment to regional lymph nodes [3,4]. In the case of our patient, we suspect tattoo pigment from her right lower extremity drained into her deep and superficial inguinal lymph nodes. Ultimately, this pigmented lymphatic fluid migrated into the external iliac lymph nodes, which was laparoscopically observed. According to the patient, the first tattoo on the right lower extremity was completed in early adulthood, and there were no obvious complications during the placement of any of her tattoos.

Migration of tattoo pigment from the dermis to lymph nodes can mimic other disease processes at the time of surgical evaluation. Many case reports have been written documenting this phenomenon, leading to incomplete sentinel or unnecessary complete nodal dissections when evaluating metastatic disease in melanoma, breast cancer, and vulvar cancer [4,6-11]. This significantly raises the risk of increased morbidity and mortality, given that it may lead to unnecessary surgical treatment and under-staging of malignancy. In the case of endometriosis, implications include missed or delayed diagnosis, increased morbidity from unnecessary tissue sampling, and confusion surrounding intra-abdominal disease processes. This has been the experience in similar surgical cases where India ink tattooing during bowel endoscopy confounds the surgeon’s findings of suspected endometriosis at the time of diagnostic laparoscopy [12,14].

On average, endometriosis is histologically diagnosed between 4 and 11 years from the onset of symptoms to surgical confirmation [15]. A delayed diagnosis can affect a patient's pain sensitivity, fertility, and mental health secondary to chronic pelvic pain. There has been a notable push towards classifying endometriosis as a clinical diagnosis, although, in more ambiguous cases of chronic pelvic pain, this can lead to unnecessary medical interventions and even prolong the time until proper treatment [15,16]. At this time, the current literature supports laparoscopy and excision of suspicious lesions for the diagnosis and treatment of endometriosis. This requires the visual identification of lesions, which has notably been shown to be inaccurate. One study included tissue sampling for 164 patients with suspected endometriosis during diagnostic laparoscopy. Of the 264 suspected endometriotic sites observed, 142 (53.8%) were confirmed histologically [16]. Significantly, 138 patients (84.1%) were found to have confirmed endometriosis.

Therefore, surgeons are at a high risk of misidentifying signs of endometriosis intra-operatively in patients where the disease process more than likely exists. This is due to the heterogeneity of lesions, inaccessible lesion locations on laparoscopy, and inter-observer variability [15]. Because of these variables, surgeons should be able to identify and safely excise any abnormal-appearing lesions in many different anatomic locations to better diagnose endometriosis. Our case suggests that reactive lymph node change from tattoo pigment migration is a potentially unexpected and misinterpreted visual cue at the time of laparoscopy. Its presence can lead to confusion, but for full evaluation and diagnosis of endometriosis, surgeons must be adequately trained and prepared to excise and rule out endometriosis in these abnormal lesions.

The strengths of this case report include its novelty, educational value, and review of the literature surrounding tattoo ink in various clinical and surgical settings. A limitation of this case report includes the fact that, although tattoo ink does confound the excision of endometriosis-like lesions on diagnostic laparoscopy, this finding is rare and does not change clinical or surgical practice. However, endometriosis has a variable appearance, and surgeons need to consider this in their differential diagnosis when doing a diagnostic laparoscopy on patients with tattoos. Additionally, given the increasing prevalence of tattoos, there may be an increased risk of tattoo ink migration and reactive lymphadenopathy. A safe biopsy of these concerning lesions is essential to completing a full evaluation and thereby preventing a false, missed, or delayed diagnosis of endometriosis. 

## Conclusions

We report a case of clinically suspected endometriosis that ultimately was most consistent with tattoo pigment lymphadenopathy. Up until now, this phenomenon has only been reported in cases of malignant melanoma, vulvar cancer, and sentinel lymph node dissection in the setting of malignancy. Moreover, it may become more frequently observed, given the increasing prevalence of tattoos among women. In gynecologic practice, this rare finding can mislead surgeons. Therefore, obtaining a thorough physical examination that includes tattoo distribution may be an important clinical consideration. Additionally, identification and safe biopsy of intra-abdominal lesions suspicious for endometriosis are important for completing an evaluation and preventing either a delayed or missed diagnosis.
